# Molecular cloning and expression characterization of flavonol synthase genes in peanut (*Arachis hypogaea*)

**DOI:** 10.1038/s41598-020-74763-w

**Published:** 2020-10-19

**Authors:** Mingyu Hou, Yongjiang Zhang, Guojun Mu, Shunli Cui, Xinlei Yang, Lifeng Liu

**Affiliations:** 1 College of Life Science, Hebei Agricultural University, Baoding, 071001 Hebei China; 2 College of Agronomy, Hebei Agricultural University, Baoding, 071001 Hebei China; 3 State Key Laboratory of North China Crop Improvement and Regulation, Hebei Agricultural University, Baoding, 071001 Hebei China

**Keywords:** Physiology, Plant sciences

## Abstract

Flavonol is an important functional bioactive substance in peanut seeds, and plays important roles responding to abiotic stress. The flavonol content is closely related to the activity and regulation of gene expression patterns of flavonol synthase (FLS). In this study, eight FLS genes, *AhFLS*s were cloned and their expression characterization in different peanut organ and seedling under different abiotic stress were conducted. The results showed that the expressions levels of *AhFLSs* were differed in all assayed peanut organs and seedlings under abiotic stress treatments. Expression levels of *AhFLS2*, *AhFLS3*, *AhFLS4*, and *AhFLS6* were higher than those of other *AhFLSs*. The flavonol contents of peanut organs and seedlings under different abiotic stress were also determined using high performance liquid chromatography (HPLC). Dried mature peanut seeds were the organ tissue with the highest flavonol content, and flavonol content increased with seed development. Under abiotic stress treatments, the types of flavonols induced differed among stress treatments. Correlation analysis results suggested that eight *AhFLS* genes may have different functions in peanut. Moreover, changes in the expression of the eight genes appear to has substrate preference. These results can lay the foundation for the study of improving nutritional value of peanut seed and resistance of peanut plant.

## Introduction

Flavonoids have medicinal effects, including antioxidant, antimicrobial, and anti-inflammatory effects, and are known to decrease the risk of certain cancers, including bladder, colon, and squamous cell cancers. Flavonoids are classified into flavonols, flavanones, flavones, isoflavones, flavanols, and anthocyanidins according to the numbers and sites of hydroxyl groups contained in the flavonoid core (diphenylchromanone)^1^. Quercetin is a flavonol that has expectorant, antitussive, anti-asthmatic, and anti-tumor effects and also slows signs of ovary aging^2^. Studies have shown that there are significant differences in the types and amounts of flavonoids found in different plant species. Leguminous crops have especially high flavonol compound contents. For example, soybeans contain isoflavone concentrations of more than 300 μg/g, and the content of quercetin in peanut seed exceeds 40 μg/g^3,4^. Peanut plants are high-quality animal feeds, and peanut seeds are a highly valued human food source.

Flavonoids participates in the regulation of multiple abiotic stress responses through its content change^5–7^. Studies have shown that flavonoids can alleviate oxidative stress in plant cells and tissues^8^. Accordingly, drought treatment increased the accumulation of flavonols in plants^9^. Flavonoid anabolism in plants under stress is regulated by jasmonic acid methyl ester (MeJA). MeJA pretreatment improved the flavonoid content of seedlings after salt stress and also enhanced the salt tolerance of seedlings^10–12^. Therefore, the characterization of the molecular regulatory mechanisms of flavonoid biosynthesis under abiotic stress and throughout seed development has substantial implications for growing of crops under complex field conditions and achieving more nutritious foods.

Plant flavonoids are synthesized by the phenylpropane metabolic pathway. Flavonol synthase (FLS) (EC1.14.11.23) is a particularly important enzyme in the metabolism of flavonols. FLS regulates the biochemical synthesis of quercetin, myricetin, and kaempferol by catalyzing related metabolic reactions^13^. For example, *Arabidopsis AtFLS1*-overexpressing strains and *Atfls1* deletion mutants altered anthocyanin anabolism and thus flavonoid content in plants^9,14^. Studies have characterized the plant gene family that includes FLS-encoding genes, revealing, for example, six gene family members in the model plant genus *Arabidopsis*^15^. However, the molecular characteristics, expression patterns of the *FLS* genes in peanut, and the regulation of the content of specific flavonol components in plants remain unclear.

In this study, the identification and characteristic of AhFLS genes under different organ and abiotic stress treatments were carried out. The contents of three flavonol components in peanut organs, seeds at different developmental stages, and seedlings under drought, salt stress, low temperature, high temperature, UV, heavy metals, and MeJA treatments were also conducted. The expression patterns of *AhFLS* in the regulation of peanut flavonol content was suggested, which provides a theoretical basis for the improvement of peanut quality through enhancements of peanut flavonol content and for future research on the functional mechanisms by which flavonols enhance abiotic stress resistance.

## Results

### Identification and characteristic of *AhFLS* genes

Based on conserved *FLS* sequences*,* such as those in *Arabidopsis thaliana*, soybean, and alfalfa, and Peanutbase data (https://www.peanutbase.org/), eight cDNAs were identified and cloned. There was a highly similar sequence on the A02 chromosome of the AA genome (*Arachis duranensis* V14167). There were four and three highly similar sequences in the A05 and A10 clusters, respectively, as well. There were two highly similar sequences on the corresponding B10 chromosomes of the BB genome (*Arachis ipaensis* K30076) (Table 1). The results of the sequence alignment indicated that the above eight *AhFLS* coding genes had two sequences with high similarity within the 150–300 base pair (bp) and 600–800 bp fragments. In this research, the aforementioned *AhFLS* genes were named *AhFLS1* through *AhFLS8*.

**Table 1:** Eight highly homologous *AhFLS* peanut sequences BLASTed against the NCBI database.

*AhFLS* gene	NCBI accession	Chromosome	Sequence identity (%)
*AhFLS1*	XM_016092605.1	A02	100
	XM_016330943.1	B02	99
*AhFLS2*	XM_016088936.1	A10	100
*AhFLS3*	XM_016112141.1	A05	97
	XM_016112140.1	A05	96
	XM_016112139.1	A05	96
	XM_016112138.1	A05	93
	XM_016344406.1	B05	91
*AhFLS4*	XM_016091208.1	A10	100
	XM_016322882.1	B10	98
*AhFLS5*	XM_016088931.1	A10	100
	XM_016326915.1	B10	99
*AhFLS6*	XM_016078871.1	A08	100
	XM_016307848.1	B07	98
*AhFLS7*	XM_016084284.1	A09	100
	XM_016318890.1	B09	98
*AhFLS8*	XM_016076468.1	A07	100
	XM_016311818.1	B07	97

**Figure 1:**
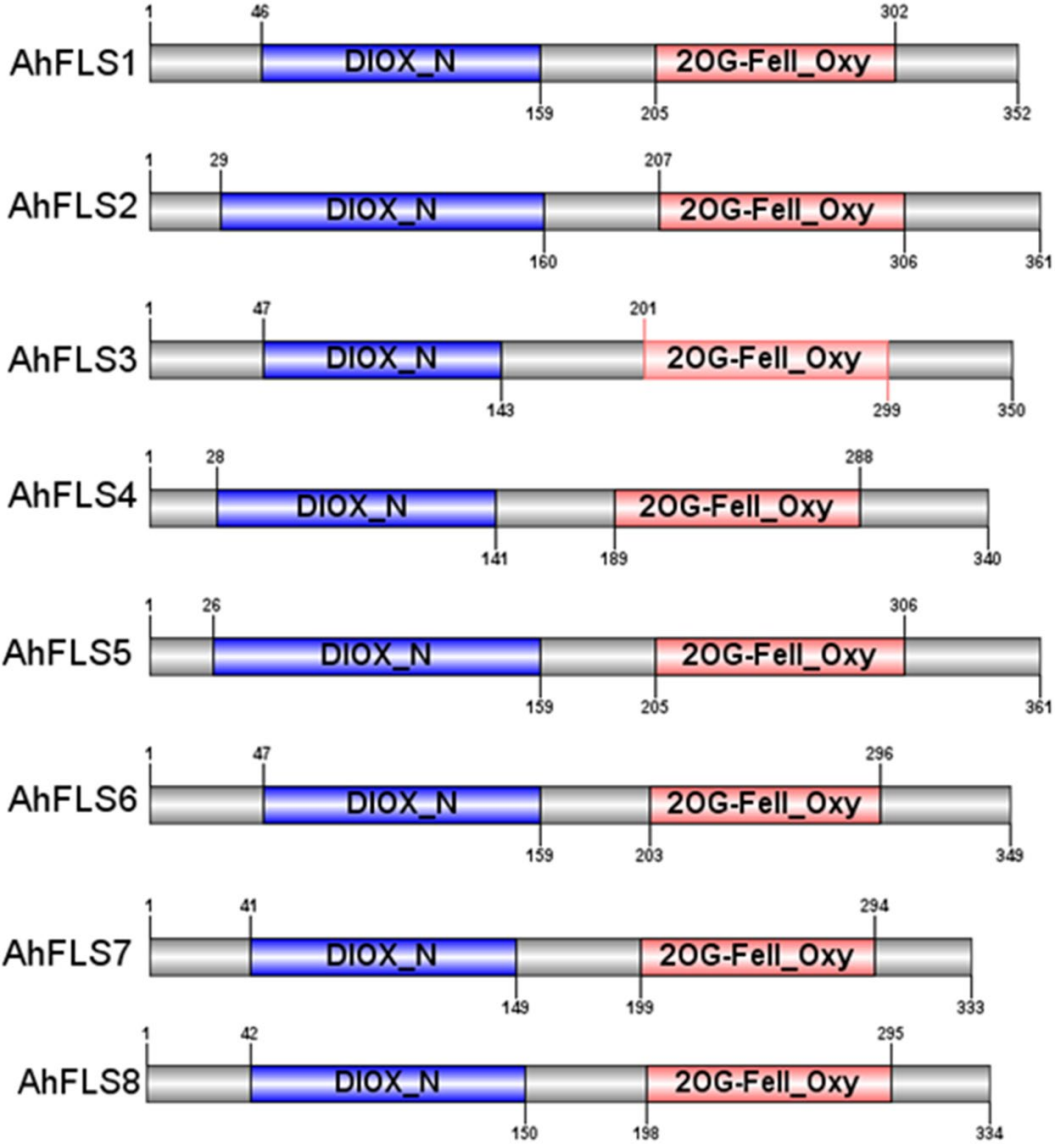
Conserved domains of AhFLS proteins. 1. DIOX_N: non-haem dioxygenase in morphine synthesis N-terminal. 2. 2OG-FeII_Oxy:2-oxoglutarate (2OG) and Fe(II)-dependent oxygenase superfamily protein.

The peptide chains encoded by *AhFLS1* through *AhFLS8* are between 333 amino acids (aa) and 361 aa in length (Table S1). The secondary structure of each protein contains α-helix, β-sheet, and random coil regions. Among them, AhFLS4 has the most α-helix regions, while AhFLS1 and AhFLS6 have the most β-sheets (Fig. S1). AhFLS3 and AhFLS4 contain transmembrane region signals (Fig. S2), but none of them are transmembrane proteins. Analysis of protein acidity and alkalinity showed that AhFLS3 is a typical basic protein (Table S1). In addition, each AhFLS protein has a distinct hydrophilic region, while the hydrophobic region is not obvious (Fig. S3). Signal P online analysis showed that the inferred AhFLS proteins did not contain significant signal peptides, but contained two conserved domains of FLS, including N-terminal DIOX_N (located at 96–133 aa) and C-terminal 2OG-FeII_Oxy (located at 93–101 aa) (Fig. 1). These structural features indicate that the tested AhFLS proteins participate in the biochemical metabolism of flavonols in peanut plants.

**Figure 2:**
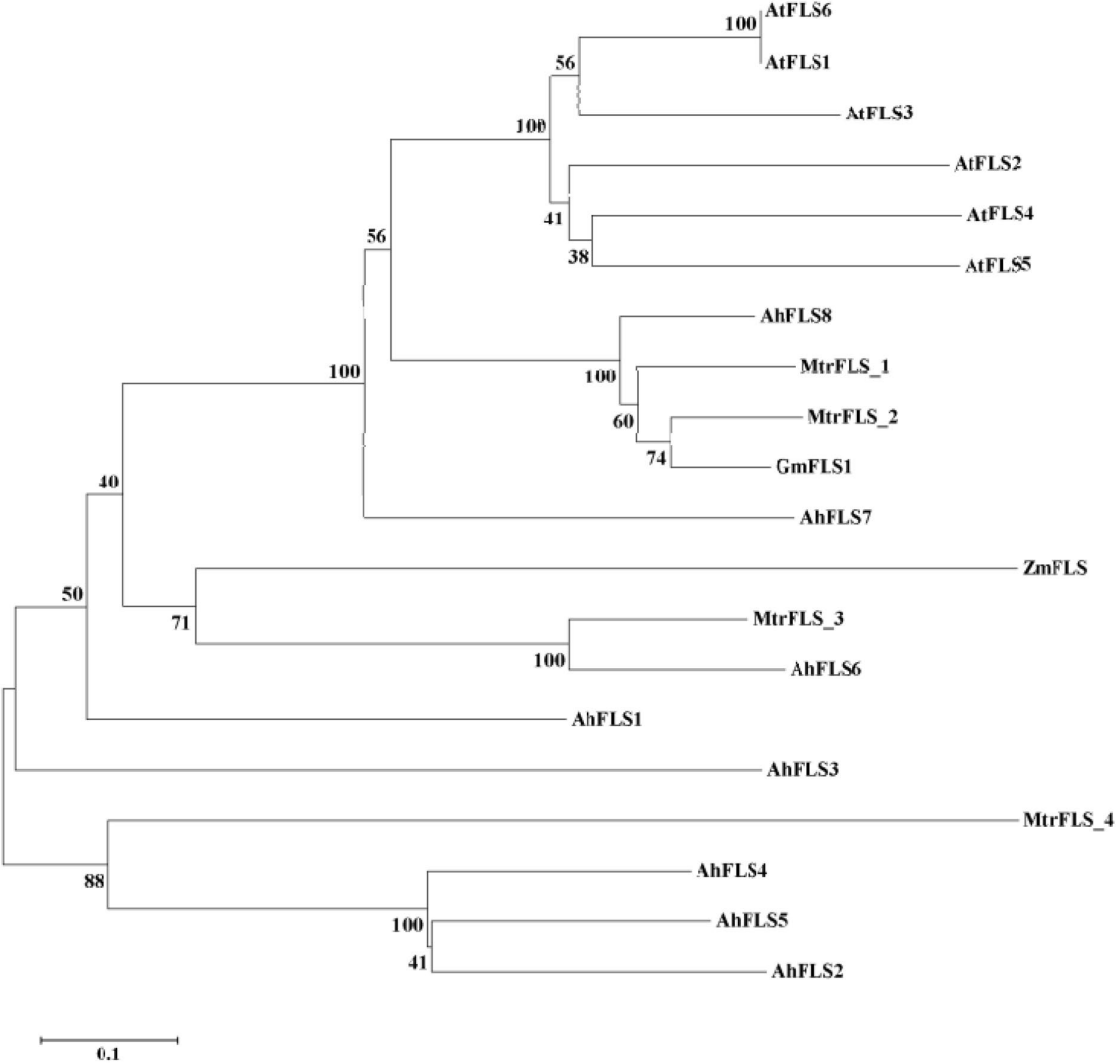
Phylogenetic analysis of AhFLS and other FLS proteins.

The gene tree of *FLS* sequences from *Arachis hypogaea*, *Arabidopsis thaliana*, *Medicago truncatula*, *Zea mays*, and *Glycine max* (Fig. 2) revealed that AhFLS2, AhFLS4 and AhFLS5 had the highest homology with other plant species’ FLS proteins. The sequences identity with MtrFLS4 was 88%. The homology of AhFLS6 to MtrFLS3 was 100%. AhFLS8 was also highly homologous to the MtrFLS1 and MtrFLS2 sequences (100% identity). Studies have shown that there is high sequence divergence between AhFLS3 and the other seven AhFLS proteins, indicating that the gene family members have been under divergent evolutionary pressures.

### Expression pattern of *AhFLS* genes among different peanut organs

**Figure 3:**
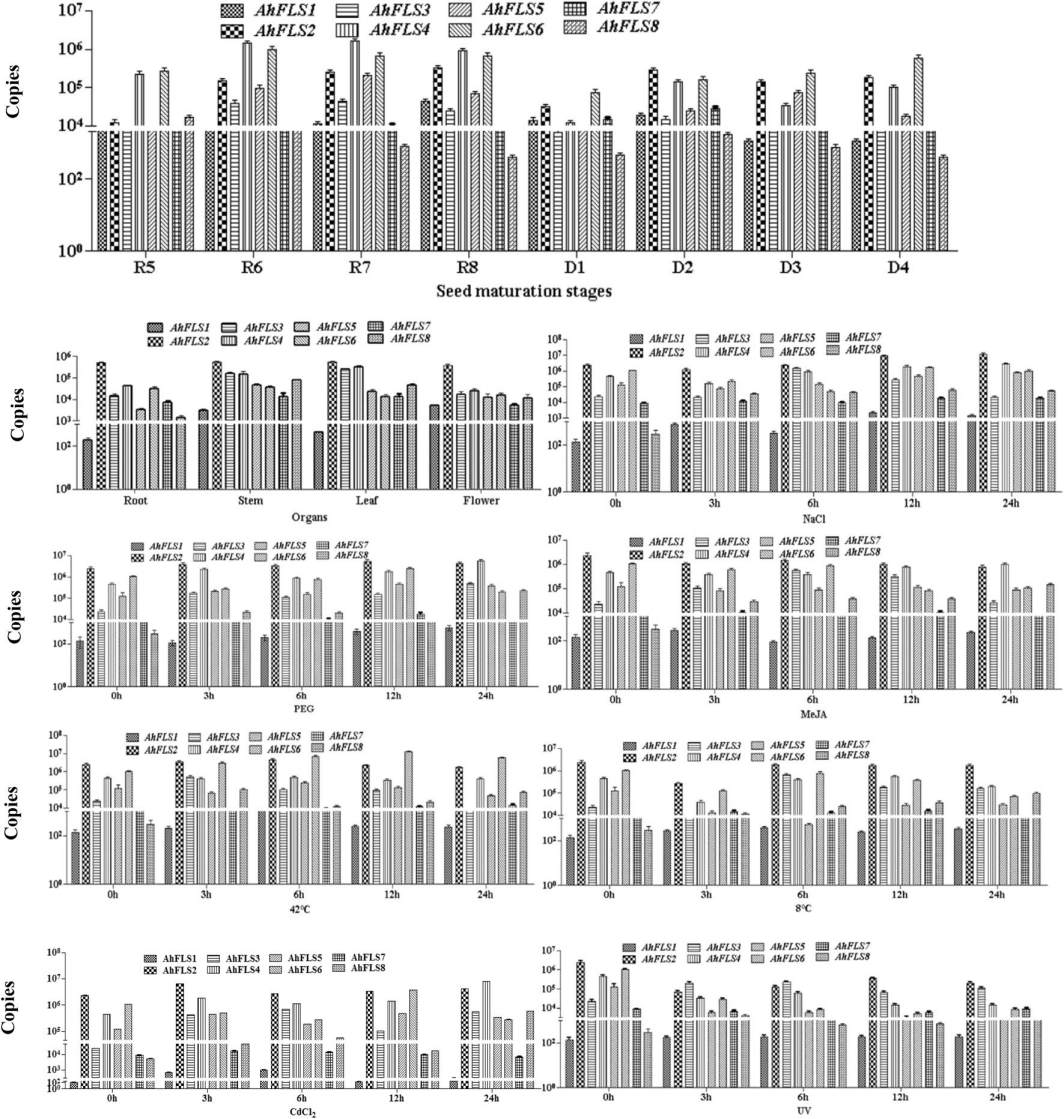
*AhFLS* expression patterns in peanut tissues throughout development and under abiotic stress. The transcription levels of genes are expressed as the copy number of the target gene in the 1 ng template, where copy number = (6.02 × 10^14^ × template concentration)/(324 × fragment length). In the formula, 6.02 × 10^23^ is Avogadro’s constant while this study expressed masses as ng. Accordingly, this value is converted to 6.02 × 10^14^, and 324 is the molecular weight of a 1 bp nucleic acid.

**Figure 4:**
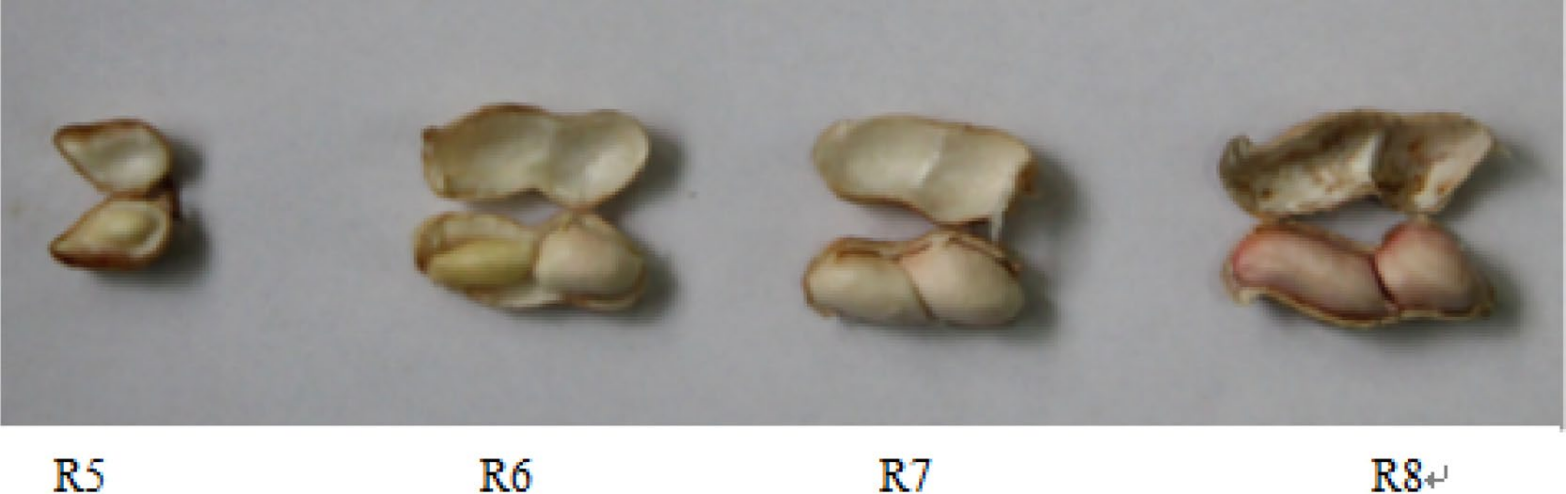
Specific reproductive growth stages for peanut cultivar ‘Yinduzhaiye’.

The expression level of *AhFLS* genes in each organ of the peanut plant are shown in Fig. 3. Among *AhFLS* genes, *AhFLS2* expression was highest in roots, stems, leaves, and flowers. During the reproductive growth period from R5 to R8, the expression of *AhFLS4* was higher than that of the other *AhFLS* genes, with the highest expression occurring when seed coat color was formed during the R7 stage (Fig. 4). The expression of this gene decreased rapidly and decreased throughout the maturation and drying processes. The expression of *AhFLS6* at the seed development stage was higher than that during the drying period.

### *AhFLS* expression patterns under abiotic stress

The varying abundance of *AhFLSs* transcripts in seedlings under different abiotic stresses is shown in Fig. 3. Compared with the test genes, the expression levels of *AhFLS2*, *AhFLS3*, *AhFLS4*, and *AhFLS6* were higher than those of the other *AhFLS* genes under various treatments. *AhFLSs* expression levels decreased over time under the 50-μM MeJA and UV treatments. *AhFLS2* expression increased after 3 h under the CdCl_2_ treatment and then decreased thereafter. Under the 42 °C, 20% PEG6000, and NaCl treatments, *AhFLS2* expression was highest at 6, 12, and 24 h after treatment, respectively. *AhFLS4* expression was higher than that of *AhFLS2* after 24 h under the CdCl_2_ and 20% PEG6000 treatments. *AhFLS6* was up-regulated by the high temperature treatment at 42 °C, reaching its highest expression at 12 h.

### Flavonol content in various peanut organs

**Figure 5:**
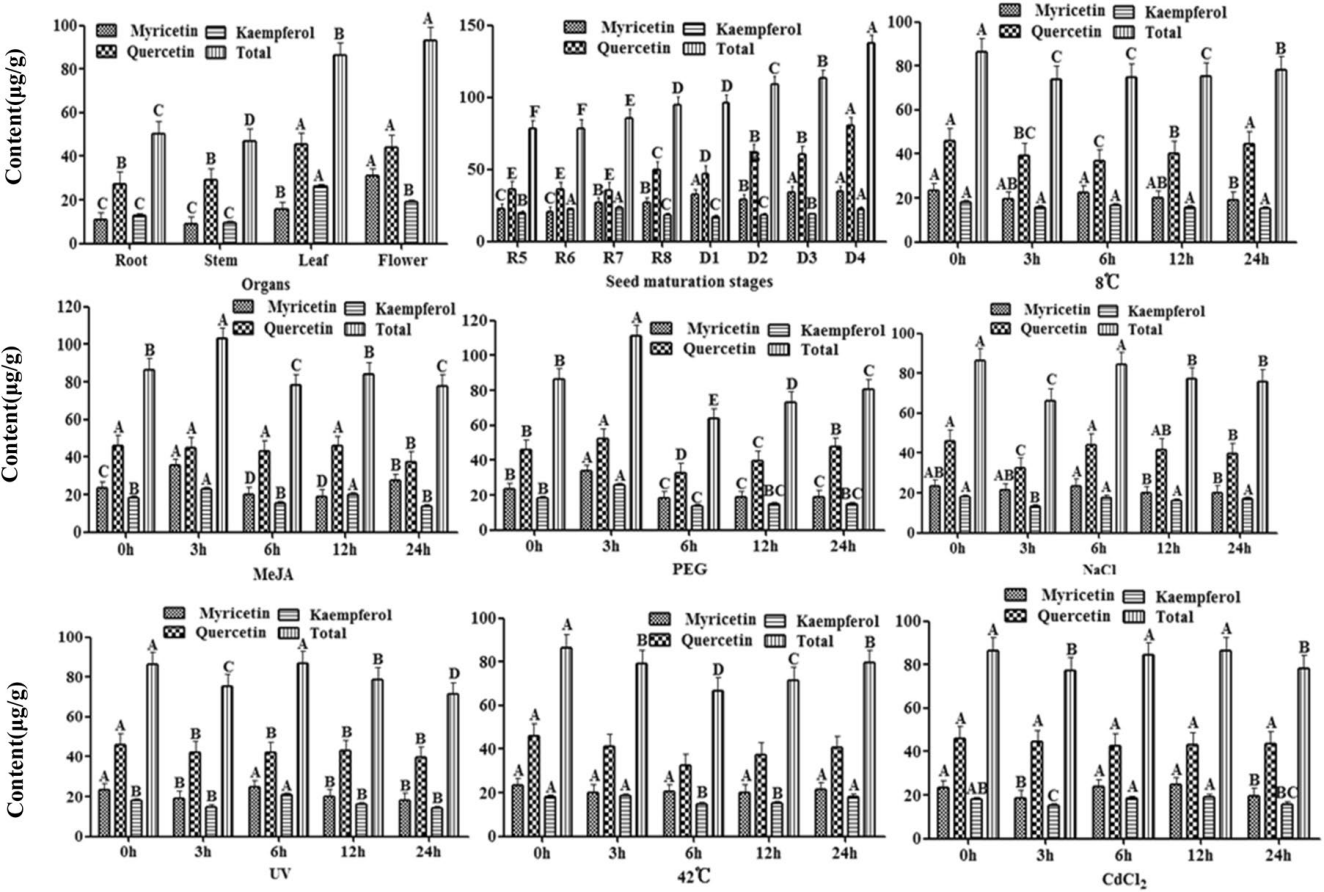
Determination of flavonol content in peanut.

Peanut plants are rich in flavonols. The combined of quercetin, myricetin, and kaempferol in stems is 46.69 μg/g fresh weight (FW), which is the lowest amount among peanut organs. The flavonol content in the reproductive organs is higher than that in the vegetative organs, especially in the seeds. The flavonol content of the seeds increased from the R5 stage, at 78.04 μg/g FW, during which the peanut seeds began to expand, to the mature D4 stage after drying, at 137.44 μg/g FW (Figs. 4, 5).

Quercetin is the main flavonol substance in various peanut organs, especially in seeds, accounting for 79% of the total flavonol content. The distribution of quercetin in organs corresponded to the total flavonol content. There were differences among organs in the percentage of the three flavonol compounds comprised of quercetin, myricetin, and kaempferol. In the vegetative organs, kaempferol content was higher than myricetin content. In root, stem and leaf of peanut plant, kaempferol contents were 12.58, 9.38, and 25.98 μg/g FW, myricetin contents were 10.41, 8.56 and 15.28 μg/g FW. In the reproductive organs, the myricetin content was higher than kaempferol content (Fig. 5).

### Effects of abiotic stress on flavonol content in peanut seedlings

The amounts of myricetin, quercetin, and kaempferol in leaves of 0-, 3-, 6-, 12-, 24-h seedlings after different abiotic stress treatments are shown in Fig. 5. Under these stress treatments, the quercetin content was the highest among the three flavonols assayed. Under the high temperature, low temperature, and NaCl treatments, flavonol content decreased first and then increased. Under PEG and MeJA treatments, the myricetin content changed significantly. Myricetin content increased to 35.21 μg/g FW and 33.63 μg/g FW at 3 h after MeJA and PEG treatments, then decreased to 18.68 μg/g FW at 12 h after MeJA treatments and 18.17 μg/g FW at 6 h after PEG treatments. Similar to myricetin content, the quercetin and kaempferol contents were also significantly regulated by PEG treatments. In addition, quercetin also showed a significant response to temperature and salt stress, and kaempferol showed a significant response to the heavy metal stress.

### Relationship between flavonol content and expression level of *AhFLSs*

**Figure 6:**
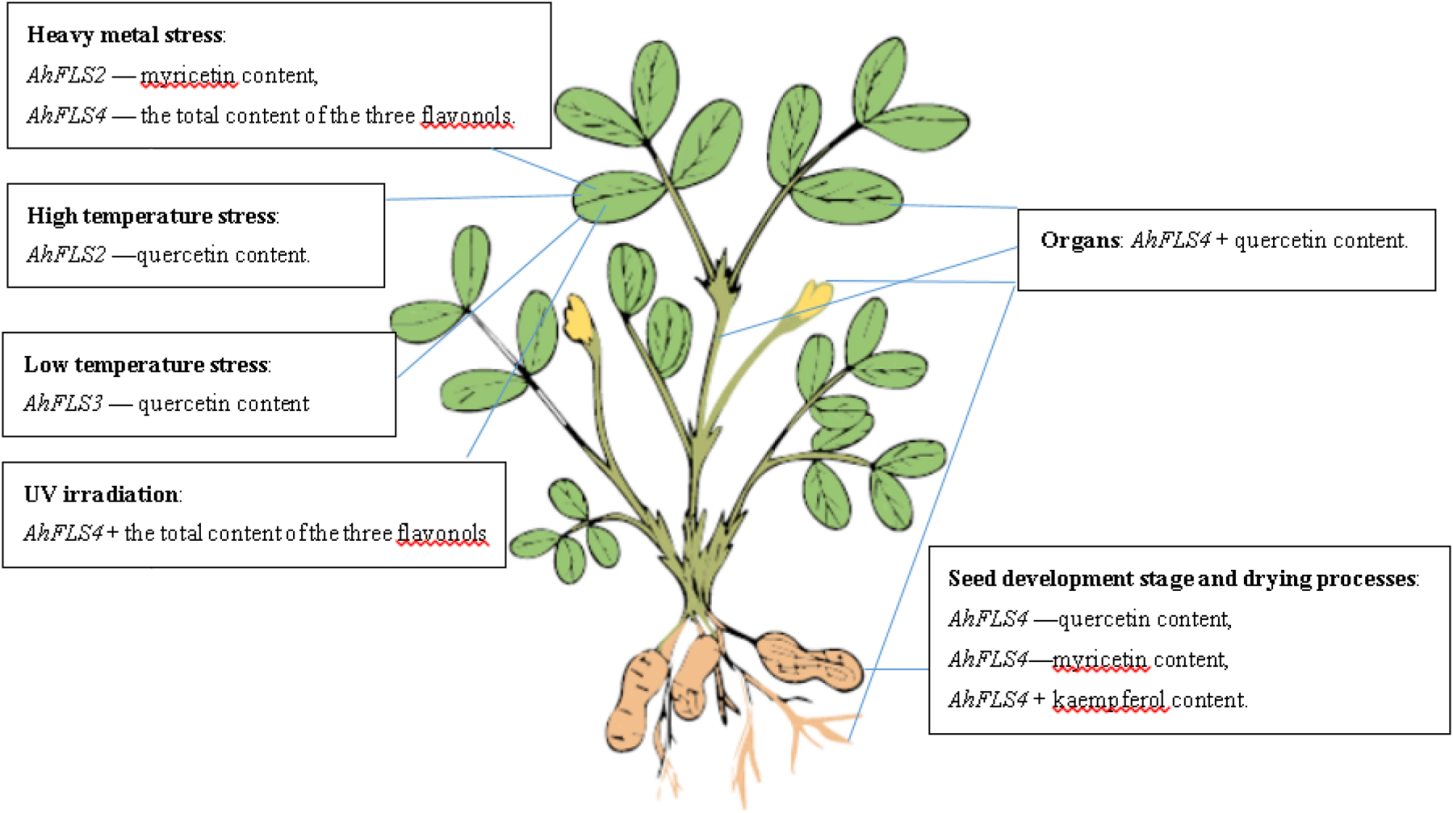
Diagram of the correlation between AhFLSs expression and flavonol content. 1. *AhFLS2, AhFLS3, AhFLS4* in figure represents its expression level. 2. “ + ” indicates positive correlation. 3. “−” indicates negative correlation.

The relationships between expression level of *AhFLS* genes and flavonol content are shown in Table S2 and Fig. 6. *AhFLS4* expression levels were highly correlated with quercetin content (*r* = 0.6577) in roots, stems, leaves, and flowers. But at the seed development stage and drying processes, the expression abundance of *AhFLS4* was highly negatively correlated with quercetin content (*r* = − 0.6203) and myricetin content (*r* = − 0.6396) and positively correlated with kaempferol content (*r* = 0.6120). It is likely that *AhFLS4* changes in substrate specificity across plant development. Studies in *Arabidopsis* indicate that AtFLS1 is more prone to using dihydrokaempferol as a substrate and produces kaempferol^9,16^. In *Scutellaria baicalensis*, *SbFLS* expression is associated with kaempferol synthesis^17^. Park^18^ isolated two AcFLS genes in onion, which favors the catalysis of dihydroquercetin, which in turn synthesizes quercetin.

Correlation analysis (Table S2) showed that under heavy metal stress, the expression of *AhFLS2* was highly negatively correlated with myricetin content (*r* = − 0.8506), and *AhFLS4* expression was highly negatively correlated with the total content of the three flavonols (*r* = − 0.6385). Under high temperature stress, the expression of *AhFLS2* was highly negatively correlated with quercetin content (*r* = − 0.6094). Under UV irradiation, the expression of *AhFLS4* was highly positively correlated with the total content of the three flavonols (*r* = 0.6155). Under low temperature stress, *AhFLS3* expression was highly negatively correlated with quercetin content (*r* = − 0.6604). These results indicate that abiotic stress treatments have important effects on the metabolic synthesis of flavonol compounds in peanut through the regulation of different *AhFLSs* expression levels.

## Discussion

### Variation in flavonol composition among plant organs

The contents and proportions of the three flavonol compounds examined—myricetin, quercetin, and kaempferol—vary among the different plant species and tissues that have been examined. It was found that the leaves and flowers of *S*. *baicalensis* contained more myricetin, while kaempferol content was highest in roots, but with quercetin as the main flavonol in stems^17^. The quercetin content in leaves of *Allium fistulosum* was higher than that of pseudostem tissue^19^. Wang et al.^3^ showed that quercetin is the main component among five flavonoids in peanut seeds, namely daidzein, genistein, myricetin, quercetin, and kaempferol. In this study, quercetin was identified as the main flavonol in peanut plants. The quercetin content in seeds gradually increased throughout seed development. The contents of the flavonols myricetin, quercetin, and kaempferol in peanut seeds after drying were higher than those of the fresh seeds, which improves the functional food value of peanut seeds.

### Variation in flavonol components in plant seedlings under abiotic stress

Flavonoids scavenge free radicals and thus play an important role in protecting cells and tissues from oxidative stress^8^. Many studies have confirmed that plant tolerance to abiotic stresses such as drought, salt, and high temperatures is related to flavonoid content. For example, when *Triticum turgidum* (L.) subsp. *turgidum* (L.) convar. *durum* (Desf.) is subjected to a high-temperature treatment during seed development, its anthocyanin synthesis is promoted, thereby improving the antioxidant capacity of the plant^20^. Similarly, the flavonoid content of grape seedlings and fruits was significantly increased by UV-B treatment^21^. MeJA treatment improved the salt tolerance of various plant species, including petunia^10^, *Medicago sativa*^11^, and soybean^12,22^, by promoting flavonoid anabolism. A low-temperature treatment at 4 °C can effectively increase the quercetin content of tobacco plants^23^. These studies show that the response of plants to abiotic stresses and specific hormones is closely related to the anabolism and content of flavonoids. In this study, the flavonol content of peanut seedlings was induced by various abiotic stresses. However, there was a difference in the degree of change in the flavonol components among myricetin, quercetin, and kaempferol. Biochemical mechanisms associated with specific stresses on the anabolic response of the aforementioned components require further investigation.

### Characteristics of AhFLS proteins

The sequences and lengths of FLS proteins are highly conserved across plant species. The approximately 330-aa-long peptide sequences contain a DIOX_N conserved domain at the N-terminus and a 2OG-FeII_Oxy conserved domain at the C-terminus. FLS proteins localize to the endoplasmic reticulum membrane and cytoplasm, and they lack signal peptides and transmembrane domains^17^. The observed characteristics of the eight AhFLS proteins in cultivated peanuts were consistent with those reported by previous studies^17^, indicating that these genes are members of the FLS family. The phylogenetic analysis of the eight AhFLS proteins showed that the sequence similarity between AhFLS3 and the other AhFLS proteins was low. However, this protein also contains DIXO_N and 2OG-FeII_Oxy conserved domains, as well as the eight aa residues conserved in the C-terminal conserved domain, which are involved in ferrous ions (His221, Asp223, and His277) binding to ketoglutarate (Arg287) and affecting protein folding (G1y68, His75, Pro207, and G1y261)^24^. The study also showed that the eight conserved amino acid residues of the tested AhFLS proteins have α-ketoglutarate-dependent dioxygenase family characteristics. The C-terminal sequences of the tested AhFLS proteins were also highly homologous. These previously mentioned protein sequence characteristics indicate that the tested peanut AhFLS proteins are involved in flavonol anabolism.

### *AhFLS* transcriptional regulation mechanisms

The *FLS* transcript levels affect the content of various flavonols in the plant, and thus participate in the regulation of plants in response to different external stresses. For example, Meng et al.^23^ showed that tobacco exhibits a significant change in the expression of specific *FLS* family members after chilling, high temperature, salt, and H_2_O_2_ stress. The present study similarly showed that *AhFLS* expression was down-regulated in peanut seedlings after UV irradiation. In addition, *AhFLS* expression patterns among different organs and various abiotic stresses revealed that *AhFLS2*, *AhFLS3*, *AhFLS4*, and *AhFLS6* were dominantly expressed genes in various tissues and organs and under different stresses. Some structural genes involved in flavonoid biosynthesis are members of larger gene families. Specific gene member expression patterns differ among growth stages, organs, and types of stress, perhaps in relation to the different regulatory elements contained by their promoter regions. Previous studies have confirmed that *GmFNSII-1* expression in soybean seedling leaves treated with MeJA decreased gradually throughout treatment, which was lower than that of *GmFNSII-2*. However, *GmFNSII-1* expression in roots and stems was higher than that of *GmFNSII-2*^12^. *MtFNSII-1* and *MtFNSII-2* are two *FNSII* members strongly expressed in *M. truncatula*. *MtFNSII-2* is highly expressed in roots under stress conditions^25^. Previous studies on the transcriptional regulation of flavonoid metabolism genes, such as *Sm4CL2*^26^, *PgD1*^27^, *VER2*^28^, and *GmFNSII-1*^12^, revealed that MeJA regulatory elements are present in the promoter regions of these genes^12^. Further identification of enhancers, especially *cis*-acting elements associated with organ- and stress-specific expression that are contained in the promoter regions of *AhFLS2*, *AhFLS3*, *AhFLS4*, and *AhFLS6*, is important for revealing the transcriptional regulation mechanism of the *AhFLS* genes.

However, *FLS* expression, flavonol content, and plant stress tolerance are not always correlated. In the flavonoid metabolism pathway, quercetin produced by FLS can be glycosylated by UFGT (UDP-glucose flavonoid 3-*O*-glucosyltransferase, EC.2.4.1.115) and rhamnosyltransferase (RT)^13,29^. High *FLS* expression may increase the content of rutin, a downstream product, while increasing the stress tolerance of plants. The accumulation of flavonols in roots of drought-treated *Arabidopsis* seedlings is associated with increased *AtFLS1* expression in this organ^9,14^. However, there were no significant differences in stress responses between a *AtFLS1*-overexpression mutant and wild-type seedlings^9^. This indicated that *AtFLS1* is a member of the *AtFLS* gene family that affects the flavonol content of *Arabidopsis* seedlings but that it is not closely related to the ability of plants to resist stress. In addition, compared to other 2-OOD enzymes in the flavonoid biosynthetic pathway, such as F3H, and ANS, FLS has a wider range of substrates. Some FLS members can convert both flavanones into dihydroflavonols and dihydroflavonols into flavonols and are thus considered to be bifunctional enzymes with both F3H and FLS activity^30^. The present study also found that although *AhFLS2*, *AhFLS3*, *AhFLS4*, and *AhFLS6* have higher expression levels under stress treatments, the expression levels of these genes were less correlated with flavonol content in vivo. This indicates that flavonol anabolism in plants under abiotic stress is not only related to the transcription of *FLS*, but is also to a large extent regulated at protein translation and post-translational levels. In this study, the expression of *AhFLS1*, *AhFLS5*, *AhFLS7*, and *AhFLS8* remained low in various organs and under stress treatments. The biological functions of the above genes in peanut tissues, organs, and the mediation of abiotic stress responses of plants merits further exploration.

## Methods

### Plant materials and treatments

Peanut seeds (cultivar ‘Yinduzhaiye’) were planted in the experimental station of College of Agronomy, Hebei Agricultural University. The roots, stems, leaves, and flowers of the plants were collected at the flowering stage and stored at − 80 °C after being quickly frozen with liquid nitrogen. The flowering stage is the coexistence period of the various organs of peanut, and the vigorous growth period of the plant, which is suitable for comparing the flavonol content of various organs. Seeds were collected at different developmental stages, which were categorized according to the pod development criteria used by Boote^31^ and Gupta et al.^32^, at R5 (28 days after flowering), R6 (40 days after flowering), R7 (55 days after flowering), and R8 (86 days after flowering) stages. Tissues were stored at − 80 °C after being quickly frozen with liquid nitrogen. The pods were harvested at 90 days after flowering and dried in an oven at 35 °C. Seeds were dried for 2, 4, 6, and 8 days and were stored at − 80 °C after being quickly frozen with liquid nitrogen. The aforementioned peanut organs and seeds were used for the determination of flavonol content and RNA extraction. The purpose of this study was to elucidate the accumulation dynamics of flavonols and the expression characteristics of *AhFLS* genes throughout peanut seed development.

Seedlings were maintained at 25 °C under a light intensity of 700 μmol photons m^-2^ s^-1^, and a photoperiod of 14 h in light and 10 h in dark. During seedling growth, Murashige and Skoog nutrient solution (MS) was provided once every 7 days. When the seedlings grew to the three-leaf stage, the following seven abiotic stress treatments were conducted: salt (250 mM NaCl), drought (20% PEG-6000), cadmium (150 μM CdCl_2_), MeJA (150 μM MeJA), low temperature (8 °C), high temperature (42 °C), ultraviolet (UV radiation, UVB 1.275 × 100 μW/cm^2^, UVC 0.014 × 100 μW/cm^2^). Salt, drought, cadmium, and MeJA treatments were implemented by supplementing MS solution with the relevant substances. Low and high temperature treatments were implemented by placing the test seedlings in a corresponding temperature incubator. The ultraviolet treatment was implemented by placing the seedlings under ultraviolet light radiation. Leaves were collected at 0, 3, 6, 12, and 24 h after treatment, and were frozen at − 80 °C by liquid nitrogen. The aforementioned samples were prepared for the determination of flavonol content and of the expression pattern of *AhFLS* genes.

### Gene cloning and sequencing

Highly conserved *FLS* sequence motifs were identified from DNA sequences of *Arabidopsis thaliana*, *Medicago truncatula*, and *Glycine max* in NCBI’s GenBank database. The peanut sequencing database (https://www.peanutbase.org) was searched for *AhFLS* coding gene sequences with the conserved *FLS* sequence. Primers used to clone *AhFLS* members were designed according to the obtained peanut *FLS* sequences (Table 2).

**Table 2:** Primer sequences used to amplify *AhFLS* sequences.

Primer name	Purpose of primers	5′-Forward	3′-Reverse
AhFLS1	Clone	GAAGTTTATTAATTTACCG	CTTAACTACAAGGCAGCTAG
	RT-qPCR	TGTTGCATAGAGCACTGGTA	ATGTGAAATTTGTGTACTTGGGA
	Standard curve	TGTTGCATAGAGCACTGGTA	ATGTGAAATTTGTGTACTTGGGA
AhFLS2	Clone	GTTGGTTAGTACCCACTGAAC	CCAGCCTTATTAAGGTGCTA
	RT-qPCR	GTCTTCAAGTTAAGCGACGAA	CACCTGCATTATGTCACCA
	Standard curve	TCTTCAAGTTAAGCGACGAA	CTTATTAAGGTGCTATCCTGA
AhFLS3	Clone	ATTACAGGTTCTAACCTTCGAG	GTTCTTATTCTATCCTATAAAATC
	RT-qPCR	ACCAGATTTATTTAGGCAG	TTTCTTTAATTTATGCACCTT
	Standard curve	ACCAGATTTATTTAGGCAG	TTTCTTTAATTTATGCACCTT
AhFLS4	Clone	CTTCTCTCTGTACCCTCTGTATC	CTCCTAATCCAAAATTCTG
	RT-qPCR	TCAGCACAAGTTCCACTCA	GCTCCAACAACTTGTATGCTA
	Standard curve	TTCAAGGACCAGCTAAGCA	GATATTCTCCAGATCACGCTTC
AhFLS5	Clone	CTAGTATTTTCTACCATCTCC	GTTGAGACTAAGTTATTGG
	RT-qPCR	TGGCTTCTTCAAACACAAC	ATTAAGCACAGTTAACACTCC
	Standard curve	TCATTATCCACCTTGCCCTT	TACTTCTCATTCTTGCAATGCG
AhFLS6	Clone	GTTGTAATCGGTACCGAAGACG	GAACTGGTATCATCAGTTAAGG
	RT-qPCR	TCCCTAATTGCCTCATTGTCA	TACAAGCACAAGGCTAATCCTG
	Standard curve	TTGCATTTGAATATAGCCGAA	GCCCAATTTCCTTATCGAG
AhFLS7	Clone	GTAAGGTATATATTACCTTCATC	CATATAATTTGTTCTATTGG
	RT-qPCR	GCCGTTGATTACCTGCAA	ATTGTAATCGTTGAGAAGTGGAG
	Standard curve	GCCGTTGATTACCTGCAA	ATTGTAATCGTTGAGAAGTGGAG
AhFLS8	Clone	CGTTTTATTATTATTACCTCC	CACGTTTAATCATTACAGAGG
	RT-qPCR	CAAGCTTAATAAGATCCCT	ATATATATGCATACATAGTTGTT
	Standard curve	CAAGCTTAATAAGATCCCT	ATATATATGCATACATAGTTGTT

Peanut RNA was extracted using the RNAiso Plus kit (Takara Biotechnology (Dalian) Co., Ltd, Dalian, China). The RNA was reverse transcribed into cDNA using the One-Step RT-PCR Kit (Takara Biotechnology (Dalian) Co., Ltd). PCR-amplified products of expected sizes were gel-purified using a SanPrep column DNA gel recovery kit (Sangon Biotech, Shanghai, China), cloned into a pMD19-T vector (Takara Biotechnology (Dalian) Co., Ltd), and transformed into *Escherichia coli* DH5α (TIANGEN Biotech (Beijing) Co., Ltd, Beijing, China). The amplicons were then sequenced at the Beijing Genomics Institute (Beijing, China).

### Multiple sequence alignments and phylogenetic and gene structure analyses

DNAStar software (DNASTAR, Madison, WI, USA) was used to predict the encoded protein sequences of the cloned *AhFLS* genes, and the AhFLS protein sequence alignment and phylogenetic analysis were performed using MEGA5.0. Physical and chemical properties and protein hydrophilicity were predicted using the ExPASy server (https://web.expasy.org). Swiss model (https://swissmodel.expasy.org/interactive), NCBI CDD (https://www.ncbi.nlm.nih.gov/cdd), Signal P4.1(https://www.cbs.dtu.dk/services/SignalP/), and TMPred (https://www.ch.embnet.org/software/TMPRED _form.html) online tools were used to predict three-dimensional structure model, conserved domains, signal peptides, and transmembrane domain features of AhFLS proteins. Schematic diagrams of conserved domains of AhFLS proteins were drawn by Illustrator for Biological Sequences (IBS) 1.0 software.

### Gene expression analysis using quantitative real-time PCR

Real-time quantitative methods were used to analyze the expression patterns of *AhFLS* genes across different stages of seed development and under various abiotic stresses. Fluorescent primers and standard curve primers of each of the tested *AhFLS* genes were amplified as shown in Table 2. The real-time PCR instrument used was the Bio-Rad CHROM4 platform (Bio-Rad Laboratories, Hercules, CA, USA), and the amplification product fluorescence data were obtained using Sequence Detector Version 1.3.1 (Applied Biosystems, Foster City, CA, USA). The level of *AhFLS* transcription was recorded as the copy number of the target sequence relative to the 1-ng template.

### Determination of quercetin, kaempferol, and myricetin contents by HPLC

The myricetin, quercetin, and kaempferol contents were determined by the HPLC method used by Wang et al.^3^, which is briefly described as follows. Ground tissues were mixed with 80% methanol containing 25% hydrochloric acid. The mixture was incubated at 80 °C for 1.5 h and then ultrasonically extracted for 30 min at 25 kHz. After extraction, the samples were centrifuged for 10 min at 10,000×*g*. The supernatant containing flavonols was filtered through a syringe with a 0.2-mm filter prior to injection into an HPLC system. Flavonol separation was performed on the Agilent 1100 LC-MSD-Trap-XCT HPLC system (Agilent Technologies, Santa Clara, CA, USA) using a C18 column. The flavonol standards were purchased from Shanghai Yuanye Biotechnology Co., Ltd. (Shanghai, China). The mobile phase consisted of A (acetonitrile) and B (water with 0.1% formic acid) solutions. The injection volume was 10 μL. A gradient elution was carried out at a flow rate 1 mL/min with a column temperature of 30 °C. The detection wave length was 360 nm. The gradient profile was programmed at 14% A solution from 0 to 8 min, 14–60% A solution from 8.1 to 20 min, 95% A solution from 20.1 to 25 min, and 14% A solution from 25.1 to 35 min. The average of three injections from each extraction was used for data collection.

### Statistical analyses

The flavonol content and gene expression results are expressed as the mean of three biological replicates. The significance of the difference between treatments and multiple comparisons was analyzed using the Student–Newman–Keuls (SNK) method in Data Processing System (DPS Version 8.50, Zhejiang University, China). The correlations between flavonol content and *AhFLS* transcription levels were analyzed using the Pearson method of Microsoft Excel. The degree of correlation is expressed by the correlation coefficient (*r* value).

## Supplementary information

Supplementary file 1
