# Supplementary material for: Molecular cloning and expression characterization of flavonol synthase genes in peanut (Arachis hypogaea)
Source: Sci Rep. 2020 Oct 19;10:17717. doi: 10.1038/s41598-020-74763-w (PMC7572378; doi:10.1038/s41598-020-74763-w)
Supplement: Supplementary file 1 [file 41598_2020_74763_MOESM1_ESM.pdf]

Molecular cloning and expression characterization of flavonol synthase genes in peanut (*Arachis hypogaea*)

Mingyu Hou[1,3], Yongjiang Zhang[2,3], Guojun Mu[2,3], Shunli Cui[2,3], Xinlei Yang[2,3] & Lifeng Liu[2,3]*

[1]College of Life science, Hebei Agricultural University, Baoding, 071001, Hebei, China.

[2]College of Agronomy, Hebei Agricultural University, Baoding, 071001, Hebei, China.

[3]State Key Laboratory of North China Crop Improvement and Regulation, Hebei Agricultural University, Baoding 071001, Hebei, China.

*email: lifengliucau@126.com

Supplementary Fig. S1 Three-dimensional structure model of AhFLSs

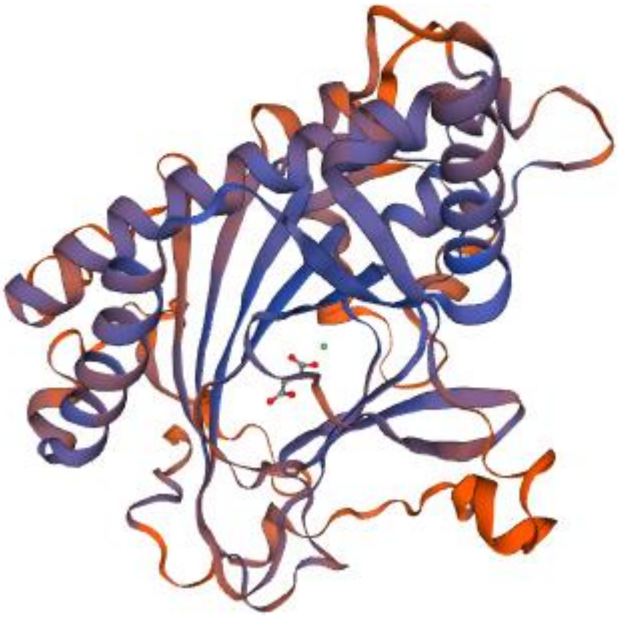

AhFLS1

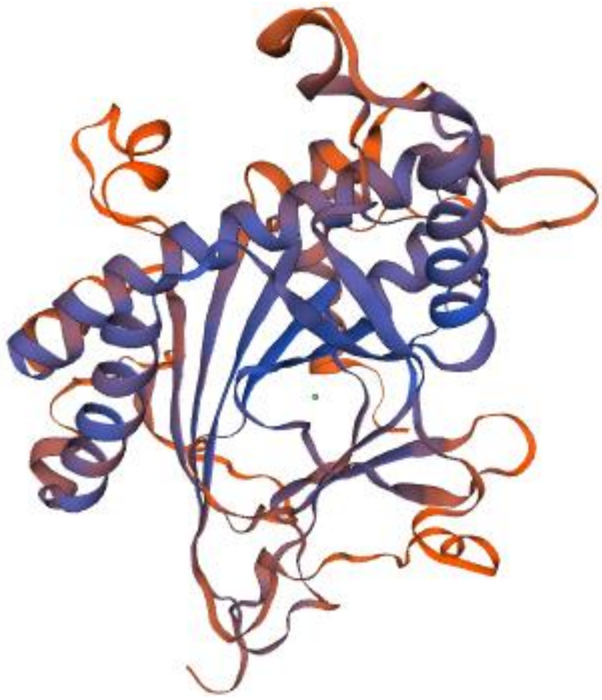

AhFLS2

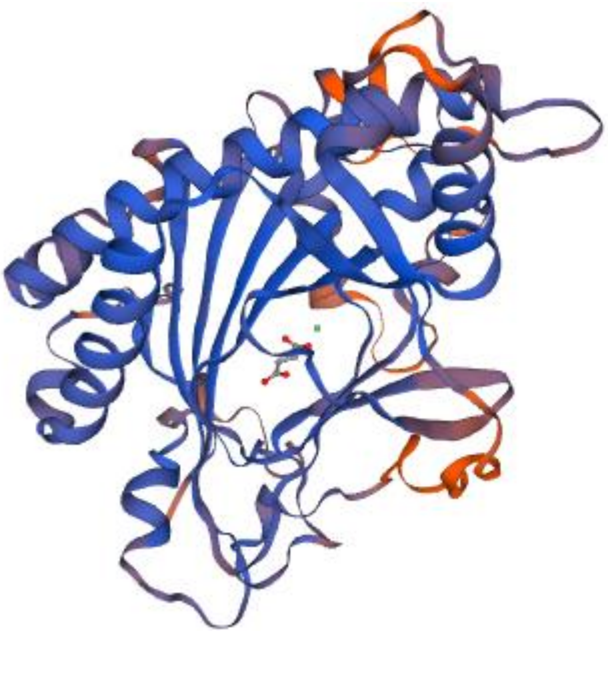

AhFLS3

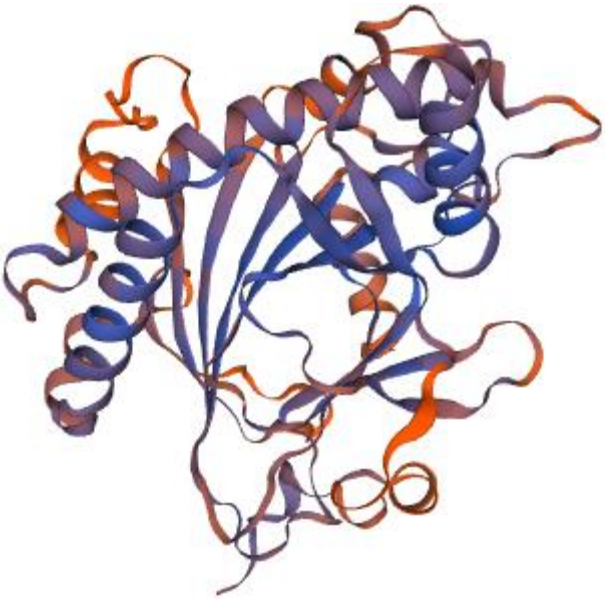

AhFLS4

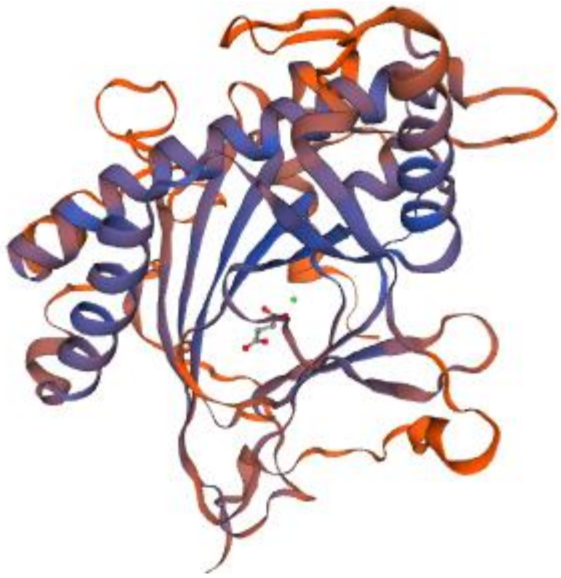

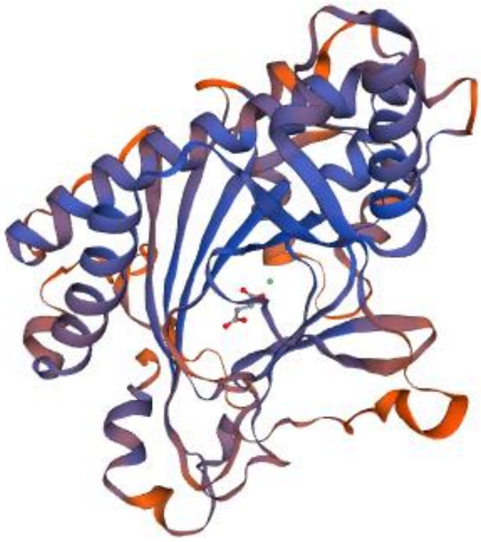

AhFLS5

AhFLS6

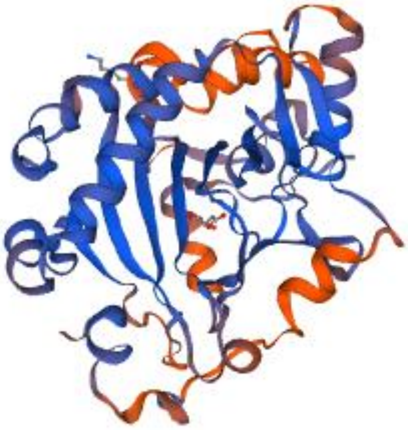

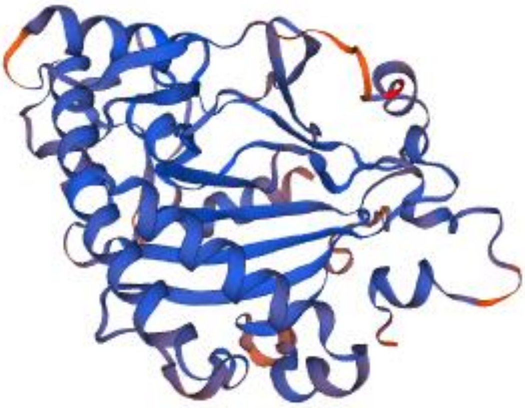

AhFLS7

AhFLS8

Supplementary Fig. S2 Transmembrane helices domains of AhFLSs

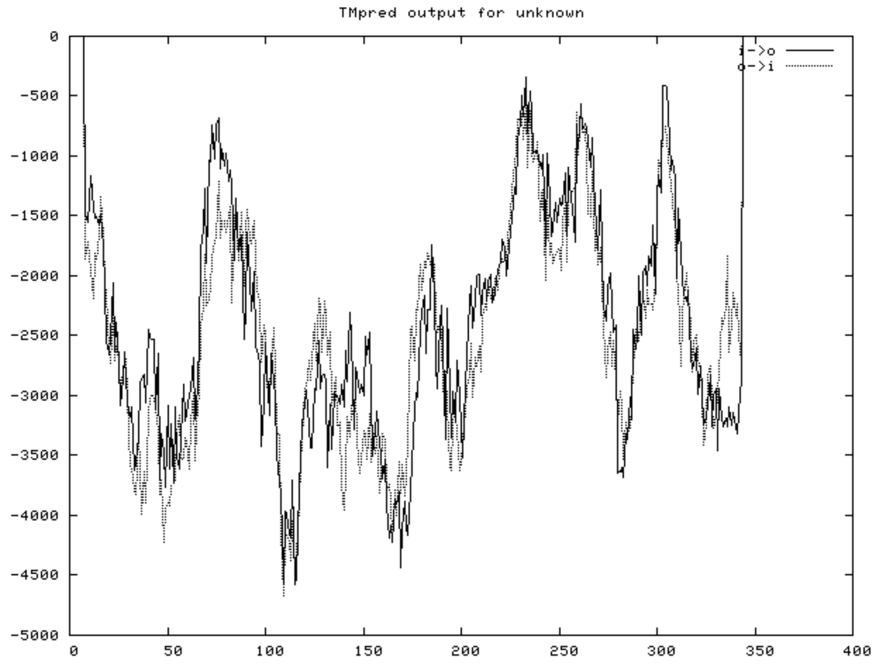

AhFLS1

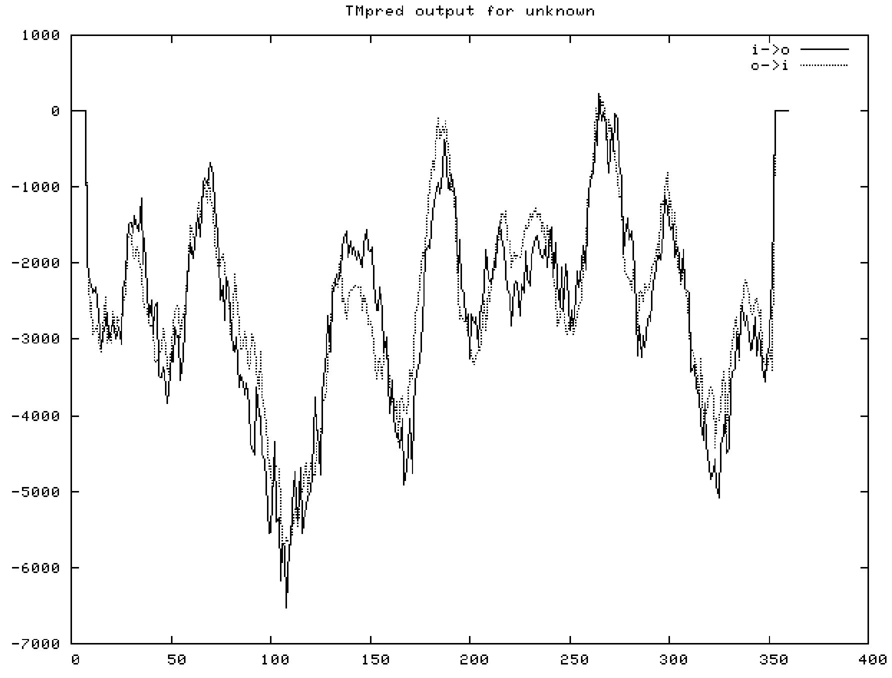

AhFLS2

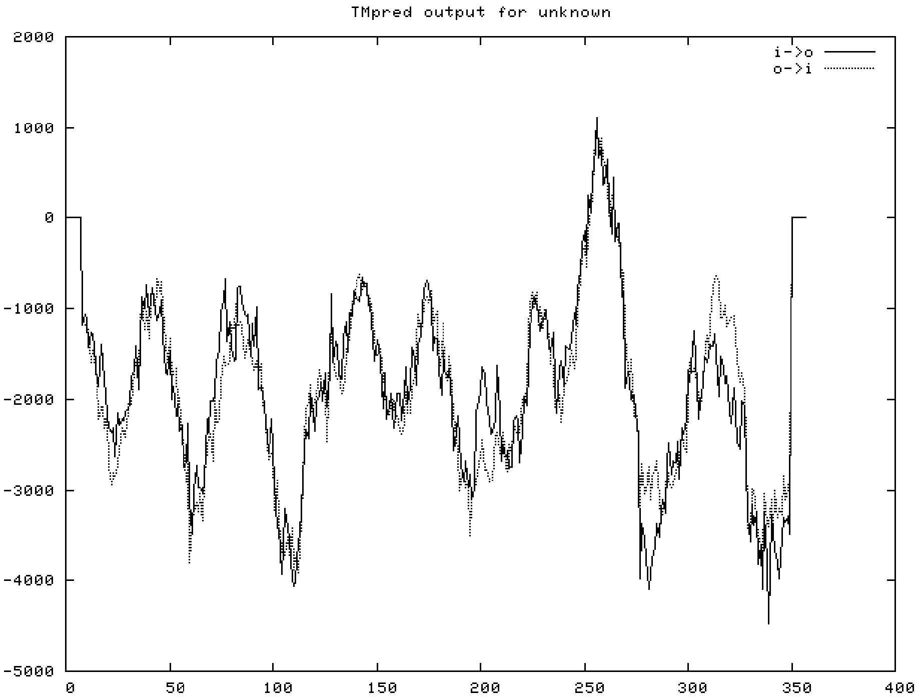

AhFLS3

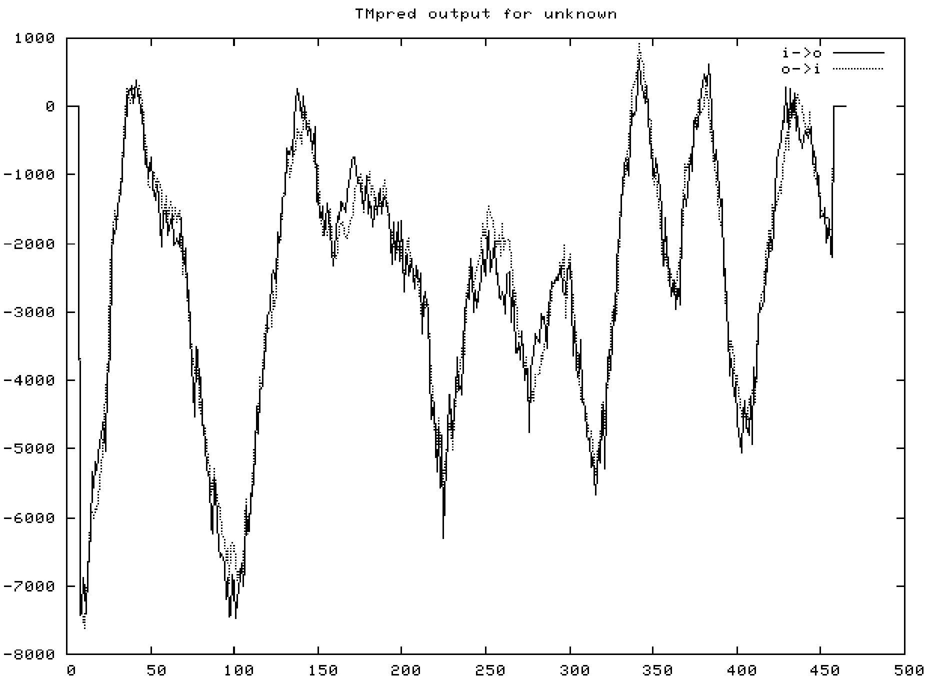

AhFLS4

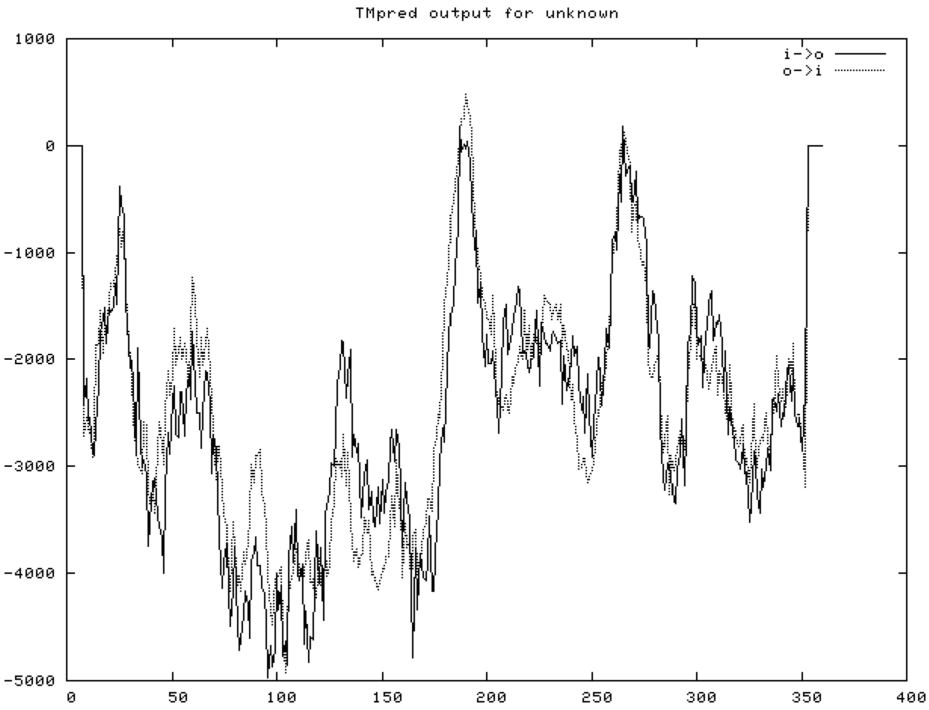

AhFLS5

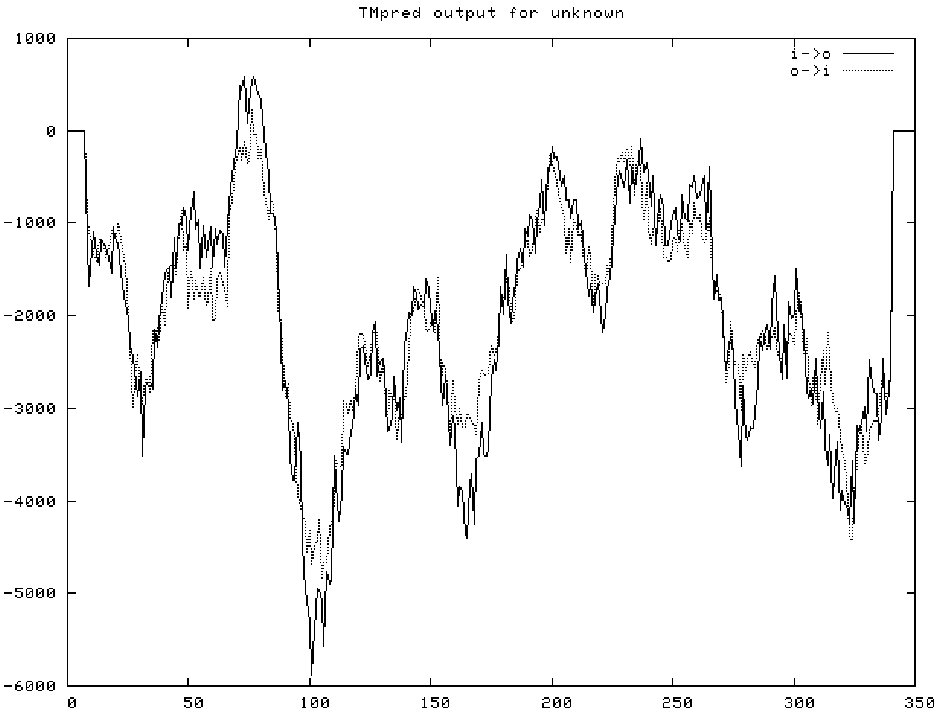

AhFLS6

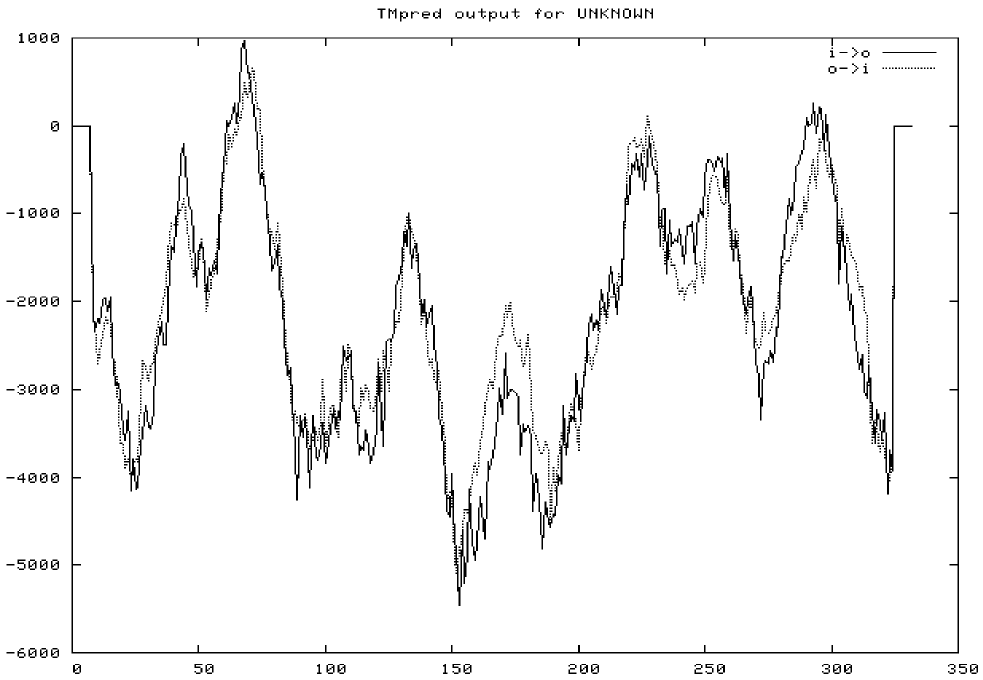

AhFLS7

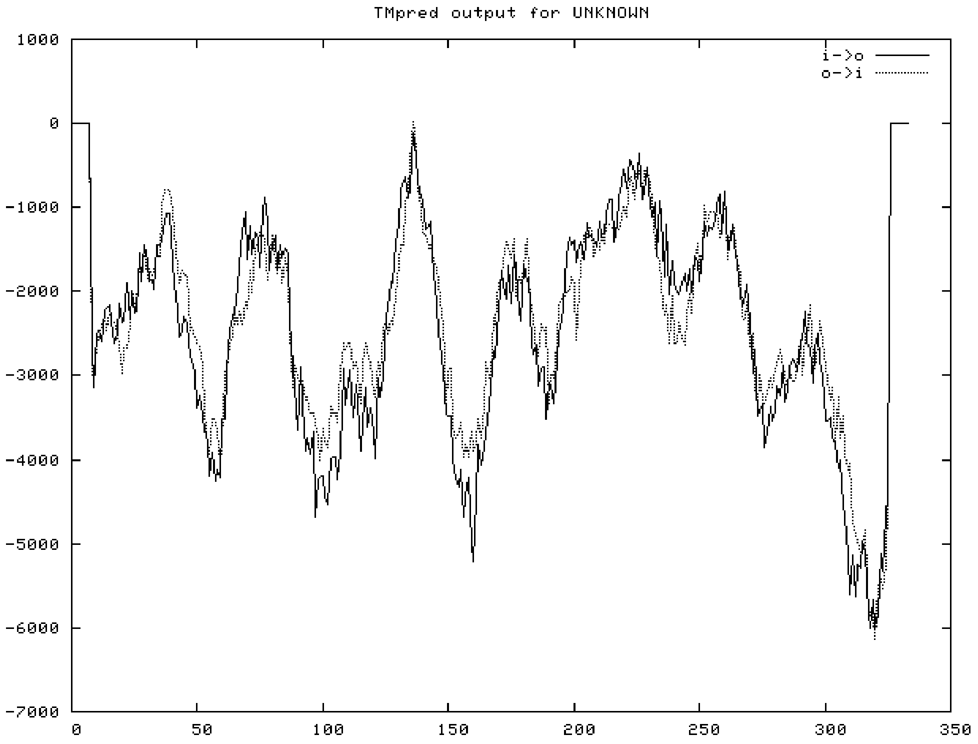

AhFLS8

Supplementary Fig. S3 Hydrophobic hydrophilic domains of AhFLSs

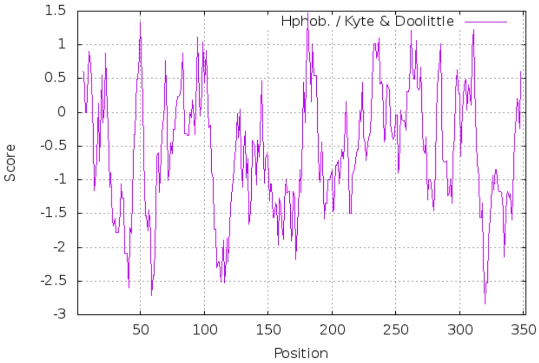

AhFLS1

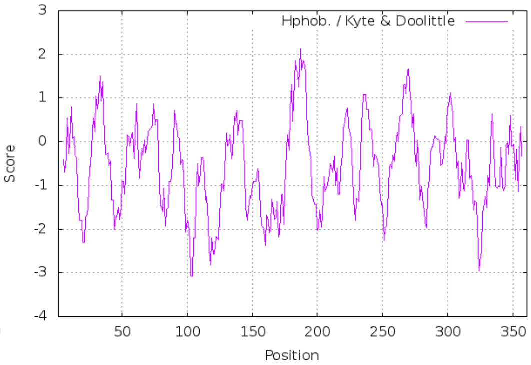

AhFLS2

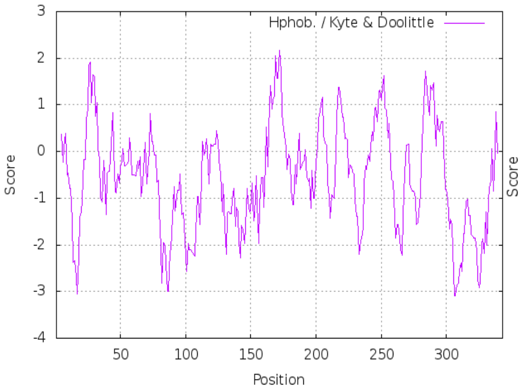

AhFLS3

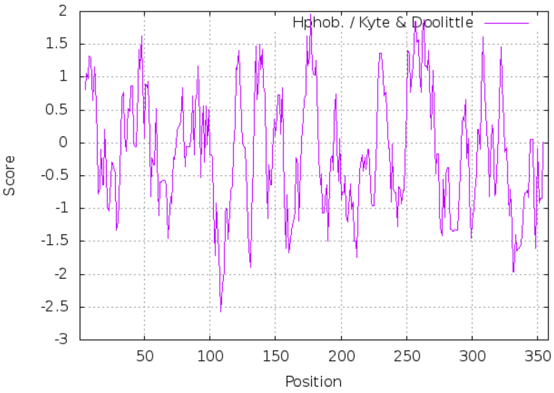

AhFLS4

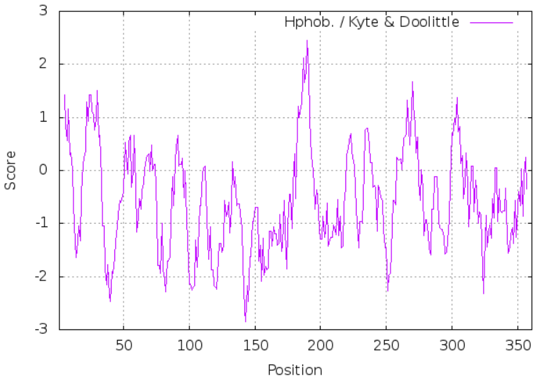

AhFLS5

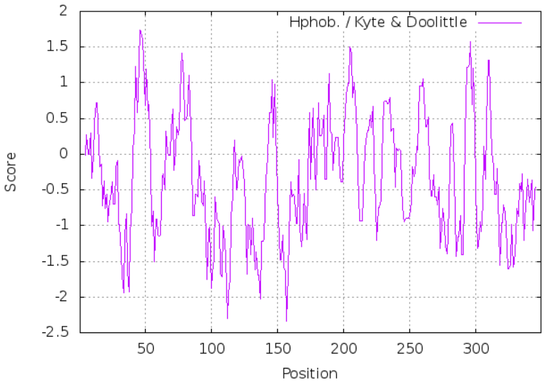

AhFLS6

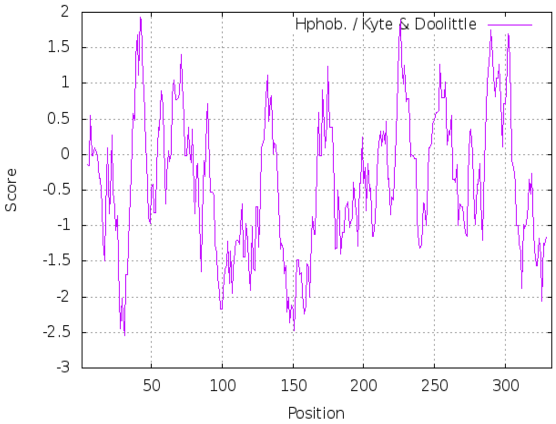

AhFLS7

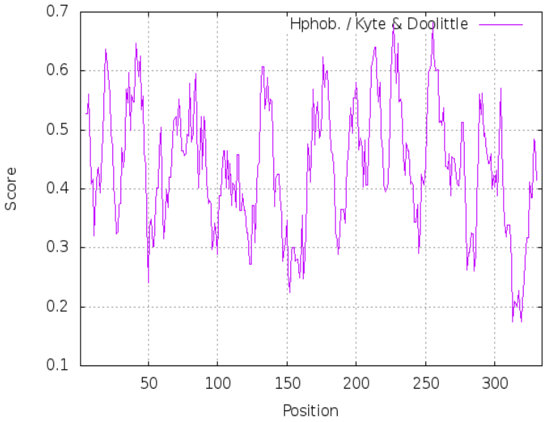

AhFLS8

Supplementary Table S1 Properties of AhFLS proteins

	AhFLS1	AhFLS2	AhFLS3	AhFLS4	AhFLS5	AhFLS6	AhFLS7	AhFLS8
**Number of amino acids**	352	361	358	343	361	349	333	334
**pI**	6.1	5.44	8.73	5.9	6.32	5.77	5.66	5.82
**Instability index**	39.31 stable	38.90 stable	50.25 （unstable）	42.63 （unstable）	45.53 （unstable）	32.74 (stable)	47.59 （unstable）	41.97 （unstable）
**Aliphatic index**	80.57	80.42	105.61	76.65	82.08	87.48	86.58	81.29
**Grand average of hydropathicity**	-0.556	-0.549	-0.140	-0.597	-0.578	-0.326	-0.468	-0.544

Supplementary Table S2 The correlation between flavonol content and *AhFLS* transcription level (*r* value)

Treatments	Flavonol	AhFLS 1	AhFLS 2	AhFLS 3	AhFLS 4	AhFLS 5	AhFLS 6	AhFLS 7	AhFLS 8
**Organs**	**Myricetin**	0.379	0.156	-0.530	-0.106	0.054	0.232	-0.273	-0.447
	**Quercetin**	0.094	0.050	0.187	0.658	0.259	0.472	-0.395	0.024
	**Kaempferol**	-0.078	-0.172	0.386	0.476	-0.123	0.115	-0.338	-0.016
	**Total**	0.191	0.042	-0.044	0.384	0.103	0.348	-0.400	-0.209
**Seed development**	**Myricetin**	-0.233	0.031	-0.597	-0.640	-0.261	-0.375	0.252	-0.676
	**Quercetin**	-0.165	0.235	-0.559	-0.620	-0.441	-0.111	0.279	-0.505
	**Kaempferol**	-0.418	0.171	0.573	0.612	0.598	0.705	-0.406	0.091
	**Total**	-0.239	0.213	-0.529	-0.583	-0.345	-0.103	0.238	-0.563
**$CdCl_2$**	**Myricetin**	-0.155	-0.851	-0.391	-0.577	-0.330	0.601	-0.223	-0.481
	**Quercetin**	-0.539	-0.002	-0.658	-0.222	-0.356	-0.122	-0.191	0.136
	**Kaempferol**	-0.114	-0.837	-0.336	-0.549	-0.295	0.604	-0.208	-0.469
	**Total**	-0.295	-0.860	-0.563	-0.638	-0.423	0.577	-0.275	-0.519
**NaCl**	**Myricetin**	-0.926	-0.810	0.514	-0.704	-0.758	-0.588	-0.967	-0.624
	**Quercetin**	-0.238	0.034	0.400	0.107	-0.003	0.283	-0.284	0.564
	**Kaempferol**	-0.272	0.154	0.377	0.266	0.176	0.194	-0.222	0.459
	**Total**	-0.412	-0.109	0.458	-0.012	-0.117	0.110	-0.439	0.368
**MeJA**	**Myricetin**	0.901	-0.278	-0.567	-0.163	-0.574	-0.020	0.415	0.095
	**Quercetin**	-0.341	0.525	0.203	-0.697	0.511	0.460	0.300	-0.967
	**Kaempferol**	0.386	-0.031	-0.146	-0.439	0.104	0.005	0.756	-0.689
	**Total**	0.606	-0.008	-0.351	-0.509	-0.157	0.150	0.648	-0.500
**PEG6000**	**Myricetin**	-0.580	-0.180	-0.180	-0.107	-0.358	-0.404	-0.472	-0.279
	**Quercetin**	-0.043	-0.002	0.304	0.410	0.041	-0.442	-0.505	0.347
	**Kaempferol**	-0.555	-0.143	-0.180	-0.098	-0.303	-0.345	-0.422	-0.270
	**Total**	-0.382	-0.106	0.015	0.109	-0.196	-0.430	-0.503	-0.030
**8℃**	**Myricetin**	-0.449	0.646	0.304	0.547	0.581	0.991	-0.359	-0.420
	**Quercetin**	-0.628	0.441	-0.660	0.074	0.803	0.097	-0.763	0.880
	**Kaempferol**	-0.702	0.513	-0.061	0.401	0.784	0.922	-0.407	-0.710
	**Total**	-0.774	0.664	-0.396	0.333	0.968	0.618	-0.780	0.999
**42℃**	**Myricetin**	-0.328	-0.376	-0.490	0.384	-0.127	-0.680	-0.084	0.217
	**Quercetin**	-0.844	-0.609	0.040	-0.202	-0.691	-0.683	-0.063	0.893
	**Kaempferol**	-0.685	-0.439	0.271	-0.112	-0.827	-0.769	0.065	0.962
	**Total**	-0.781	-0.575	0.006	-0.093	-0.678	-0.757	-0.041	0.926
**UV-B**	**Myricetin**	-0.362	0.410	0.091	0.509	0.450	0.425	-0.608	-0.457
	**Quercetin**	-0.876	0.845	-0.520	0.849	0.852	0.841	0.059	0.057
	**Kaempferol**	-0.160	0.223	0.226	0.325	0.260	0.234	-0.737	-0.501
	**Total**	-0.500	0.534	-0.045	0.616	0.567	0.543	-0.518	-0.417
**All stress treatment**	**Myricetin**	-0.200	-0.162	-0.081	-0.104	-0.118	-0.118	-0.252	-0.139
	**Quercetin**	-0.338	-0.012	0.099	0.206	0.049	-0.339	-0.308	0.056
	**Kaempferol**	-0.227	-0.059	0.016	-0.066	-0.042	-0.100	-0.221	-0.169
	**Total**	-0.311	-0.091	0.018	0.037	-0.038	-0.241	-0.317	-0.079

Note: 1. "+" indicates positive correlation; "-" indicates negative correlation. 2. 0.8-1.0 indicates extremely high correlation; 0.6-0.8 indicates high correlation; 0.4-0.6 indicates moderately correlated; 0.2-0.4 indicates weak correlation; 0.0-0.2 indicates very weak or no correlation.
